# Deep Learning Approach for Vibration Signals Applications

**DOI:** 10.3390/s21113929

**Published:** 2021-06-07

**Authors:** Han-Yun Chen, Ching-Hung Lee

**Affiliations:** 1 Department of Mechanical Engineering, National Chung Hsing University, Taichung City 402, Taiwan; Hamilton.HY.Chen@auo.com; 2 AU Optronics Corporation, Taichung 407, Taiwan; 3 Department of Electrical and Computer Engineering, National Yang Ming Chiao Tung University, Hsinchu City 300, Taiwan; 4 Department of Electrical and Computer Engineering, National Chiao Tung University, Hsinchu City 300, Taiwan

**Keywords:** vibration signal, deep learning, convolutional neural network, hyper parameter, optimization, short time Fourier transform

## Abstract

This study discusses convolutional neural networks (CNNs) for vibration signals analysis, including applications in machining surface roughness estimation, bearing faults diagnosis, and tool wear detection. The one-dimensional CNNs (1DCNN) and two-dimensional CNNs (2DCNN) are applied for regression and classification applications using different types of inputs, e.g., raw signals, and time-frequency spectra images by short time Fourier transform. In the application of regression and the estimation of machining surface roughness, the 1DCNN is utilized and the corresponding CNN structure (hyper parameters) optimization is proposed by using uniform experimental design (UED), neural network, multiple regression, and particle swarm optimization. It demonstrates the effectiveness of the proposed approach to obtain a structure with better performance. In applications of classification, bearing faults and tool wear classification are carried out by vibration signals analysis and CNN. Finally, the experimental results are shown to demonstrate the effectiveness and performance of our approach.

## 1. Introduction

Vibration signals can be applied for machine diagnosis and help discover problems during machining. By the signal processing methods, the signals can be decomposed and transformed into different domains for analysis, e.g., fast Fourier transform, wavelet transform, etc. [1,2,3,4,5,6,7,8]. Statistical features and other characteristics related to physical phenomena are then extracted for applications. Based on data analysis, machine learning approaches model the relationship of features and physical phenomena. The corresponding features are usually extracted by statistical analysis in time and frequency domains.

In mechanical systems, rolling element bearings (REBs) are one of crucial components and the bearing failures can cause safety problems. A lot of the literature has proposed the diagnosis of bearings or building monitoring systems with machine learning models, e.g., support vector machines (SVMs), neural networks (NNs) [9,10,11,12,13,14]. Recently, deep learning approaches were proposed to auto extract the characteristics of vibration signals for signals analysis [9,12,13,14]. For signals analysis, methods of frequency spectra can also be used for prediction or diagnosis [15,16]. The statistical features are usually utilized to be inputs of machine learning for diagnosis model development [17,18,19]. Herein, the convolutional neural network (CNN) discussed in this paper is also widely applied for bearing diagnosis using raw signals or spectra of signals [20,21,22,23,24,25,26].

The condition of machine tools affects the quality and the productivity directly. A blunt tool can cause terrible quality since the magnitude of vibration during machining increases. Excessive tool wear can even lead to tool breakages. The diagnoses of tool status were proposed by on-line and off-line monitoring [27,28,29,30,31]. For off-line monitoring, the tools are dismounted to measure the worn area. However, the machines need to stop in order to measure tool wear. In the on-line approach, the status of a tool can be predicted using vibration, acoustic emission, and force signals of vises and machine tools [27,28,29]. Due to the improvement of photographic techniques, on-line monitoring can also be implemented using high speed cameras in some machines [30,31]. In addition to the status of machines, predicting the quality of products is a valuable topic for the industries. If the quality can be estimated, the whole manufacturing process can be controlled easily. Predicting quality using machining parameters is discussed in many studies. Machine learning algorithms are applied to model the relation between machining parameters and quality; for instance, fuzzy logic [32], response surface methodology [33], etc. The main disadvantage of using machining parameters is that the statuses of tools and machines are not considered. Since vibrations affect quality, the vibration signals can be analyzed and applied to estimate quality [2,34,35]. Sensor fusion has also been proposed in other studies; for instance, multiple vibration sensors [36,37,38], vibration with acoustic signals [39,40] or load cell [41], etc. The sensors can be seen as evidence for fault detection. In other words, different types of sensors can provide different symptoms when the components fail. Fusion in feature domain and frequency domain are also discussed in other studies [42,43].

Deep learning approaches provide automatic feature extractions; for instance, a convolutional neural network (CNN) [40]. Applications of a CNN in vibration signals are discussed in lots of research, including bearing faults diagnosis, tool wear classification and machining roughness estimation. By employing convolutional operation, the features can be extracted automatically [44,45,46,47,48]. One-dimensional CNNs (1DCNN) and two-dimensional CNNs (2DCNN) are used in the domain of REB signals prediction. For 1DCNN applications, the inputs are raw signals or other one-dimensional data [20,25]. If 2DCNN is utilized, the inputs should be chosen as time-frequency spectra or other two-dimensional data or images [21,26,49,50].

In this study, CNNs for vibration signals analysis are discussed. Firstly, 1DCNN with sensor fusion in parallel structure is introduced for machining roughness estimation. The model structure (hyper parameters) optimization of the CNN is proposed by experimental design, data acquisition, neural network modeling, and particle swarm optimization. Subsequently, CNNs for bearing faults classification and tool wear classification are discussed later. According to the results of applications, the conclusions for utilizing CNNs in vibration signals analysis can be presented.

In the rest of paper, the applied techniques are introduced in Section 2, prediction using CNNs and structure optimization are introduced in Section 3, CNNs for classifications are discussed in Section 4, and the conclusion of the study is presented in Section 5, finally.

## 2. Theoretical Background

Herein, techniques utilized in the study are introduced, including short-time Fourier transform, convolutional neural networks and particle swarm optimization.

### 2.1. Convolutional Neural Network (CNN)

**Figure 1:**
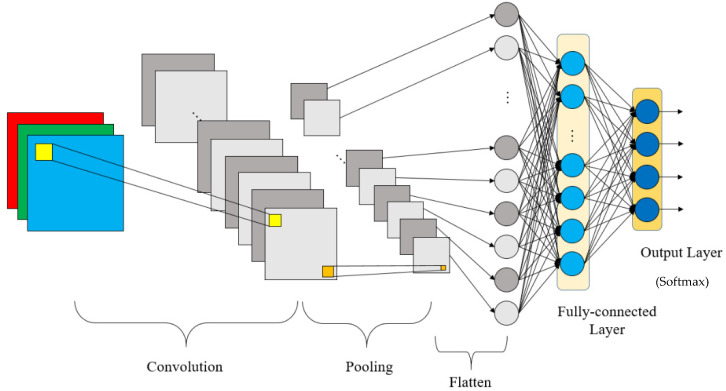
Structure of convolutional neural network. Reprinted from ref. [47].

The CNN was first proposed by Lecun et al. [51] and the structure of the CNN is shown in Figure 1. The three basic operations in the CNN are convolutional layers, pooling layers, and fully connected layers. Convolutional layers and pooling layers are adopted for automatic feature extraction when fully connected layers are general neural networks which play the roles of classifier or predictor.

At first, the convolutional layer is introduced, and the inputs are convolved by filters to obtain the corresponding features. The convolutional operation of single filter can be represented as

(1) $$z_{l}^{k}=f_{c}\left(\alpha_{l}*x+b\right)$$

where * represents the convolutional operation; $x\in {ℛ}^{W\times L}$ denotes the input and ***f*_c_** denotes the activation function of convolution layer; *b* and $\alpha_{l}$ are the bias and corresponding kernel of the lth filter, respectively; $z_{l}^{k}$ denotes the corresponding output feature map. Herein, kernel matrix $\alpha_{l}$ are obtained by training and *l* = 1, …, *N* is the selected kernel size.

In pooling layers, the important features are reserved, and the number of features are reduced by a max-pooling operation. The operation of a single filter can be represented as

(2) $${p_{l}^{k}}_{q,r}=\max\left(\left[\begin{array}{ccc} {z_{l}^{k}}_{q,r} & \begin{array}{cc} {z_{l}^{k}}_{q,r+1} & \ldots \end{array} & {z_{l}^{k}}_{q,r+L_{P}}\\ \begin{array}{c} {z_{l}^{k}}_{q+1,r}\\ \vdots \end{array} & \ddots & \begin{array}{c} {z_{l}^{k}}_{q+1,r+L_{P}}\\ \vdots \end{array}\\ {z_{l}^{k}}_{q+W_{P},r} & \begin{array}{cc} {z_{l}^{k}}_{q+W_{P},r+1} & \ldots \end{array} & {z_{l}^{k}}_{q+W_{P},r+L_{P}} \end{array}\right]\right)$$

where q and *r* are the row and column index of features after pooling, $L_{P}$ and $W_{P}$ represent the length and width of filters in pooling layers.

The feature maps after feature extraction are flattened into a one-dimension array and inputted into fully connected layers. The feedforward operation of a single neuron in fully connected layers is represented as

(3) $$y=f_{f}\left(\overset{n}{\underset{a=1}{\sum }}w_{a}h_{a}+b\right)$$

where $h_{a}$ is the input of the neuron, $w_{a}$ is weight of $h_{a}$, a=1, 2, …, n, *b* is the bias, $f_{f}$ is the activation function of the neuron in the fully connected layer, *y* is the output of the CNN.

### 2.2. Short-Time Fourier Transform (STFT)

Discrete Fourier transform (DFT) is widely applied to generate frequency spectra of signals. However, frequency spectra do not contain the information of time domain. In order to present time domain and frequency domain at the same time, STFT is employed [8,52]. In STFT, signals are divided into short-time segments firstly, and frequency distributions of segments are computed by DFT. Finally, the time-frequency spectra of signals can be obtained by stacking the frequency spectra of segments. STFT can be represented as

(4) $$\mathrm{STFT}\left(x\left[n\right]\right)\equiv X\left(m,\;e^{-j\omega }\right)=\overset{N-1}{\underset{n=0}{\sum }}x\left[n\right]w\left[n-m\right]e^{-j\omega n}$$

where *x* is the discrete signal with size *N*, ω is frequency, n is the index of data points in *x*, *w* is discrete window function, *m* is discrete index in the window *w*. STFT is applied as the preprocessor of signals in the study. The time-frequency spectra are the inputs of convolutional neural networks, which is introduced in the following section. Note that the axes of spectra are removed when input into the model.

### 2.3. Particle Swarm Optimization (PSO)

Particle swarm optimization (PSO), simulating the social behaviors of fish and birds while foraging, was proposed in 1998 [53]. Firstly, the fitness function and the target of optimization are defined. By fitness function, the score of particles can be evaluated. The particles adjust their directions and locations according to the best location of the group and themselves using

(5) $$V_{i}\left(t+1\right)=w\times V_{i}\left(t\right)+\mathrm{random}\times c_{1}\times \left(P_{pbest}-P_{i}\left(t\right)\right)+\mathrm{random}\times c_{2}\times \left(P_{gbest}-P_{i}\left(t\right)\right)$$

and

(6) $$P_{i}\left(t+1\right)=P_{i}\left(t\right)+V_{i}\left(t+1\right)$$

respectively, where $V_{i}$ is the direction of the *i*th particle, *t* represents the index of iteration, *w* is the weight of inertia, $c_{1}$ is the weight representing how much $P_{pbest}$ affects the optimization, $c_{2}$ is the weight representing how much $P_{gbest}$ affects the optimization, $P_{i}\left(t\right)$ represents the location of the *i*th particle at the *t*th iteration. Finally, while reaching the set maximum of the iteration or the fitness of $P_{gbest}$ remains the same, the optimization is complete and $P_{gbest}$ is the optimized result. In this study, the minimized mean absolute percentage error (MAPE) of prediction is adopted to be the objective function for optimization of hyper parameters.

## 3. Machining Roughness Estimation Application

In this section, machining surface roughness estimation is achieved using the CNN. The optimization of the CNN structure is also discussed. Firstly, the dataset is introduced. Then, the experimental design is carried out and executed. After the experiments are complete, a simple neural network (NN) is applied to model the relation between hyper parameters and the performance of model. Optimization using PSO is then discussed. The optimized results are verified, finally.

At first, the optimization of the model structure is introduced.

### 3.1. Optimization of Model Structure

**Figure 2:**
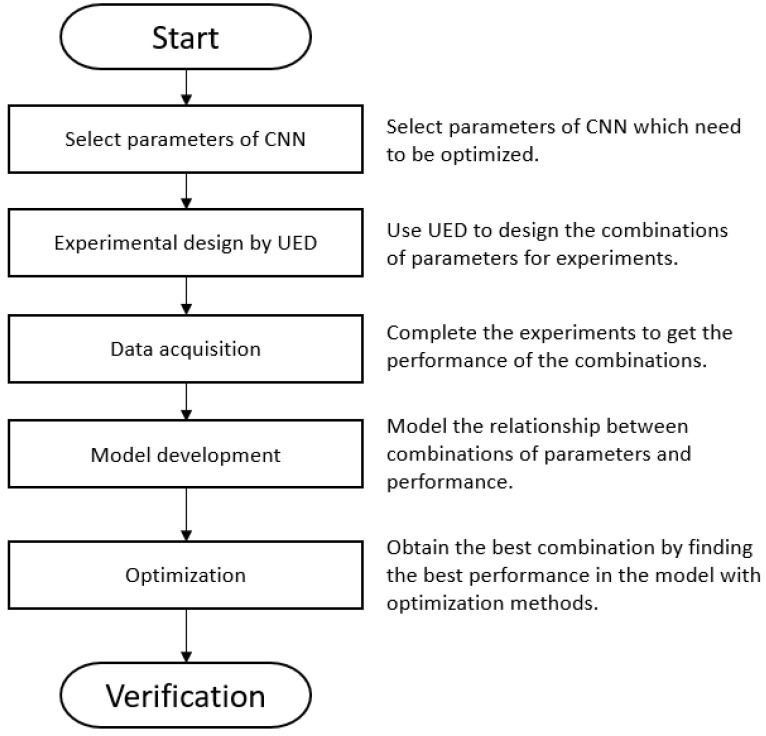
Flow chart of the proposed optimization procedure.

Herein, the concept of optimizing the model structure (hyper parameters) is utilized [54]. An improvement by uniform experimental design (UED) [55], a neural network, and a PSO algorithm is introduced. It preserves the ability of the CNN and optimizes the performance. The procedure of optimization is introduced. The flow chart of optimization procedure is shown as Figure 2. The procedures include (1) parameter selection of the CNN, (2) experimental design using UED, (3) data acquisition, (4) model development, (5) optimization, and finally, (6) validation.

#### Optimization Procedure

Step 1. Parameter selection of CNN: Select the main structure (convolution filter size, pooling, fully connected nodes), the optimized hyper parameters, and levels. 

Step 2. Design experiments using UED: Choose the appropriate uniform layout (UL) of model structure according to the parameter selection and design experiments.

Step 3. Data acquisition: Complete the experiments. The model with the above structure is trained and the corresponding hyper parameters/trained MAPE are collected as input/output data. 

Step 4. Model development: Modeling the function between hyper parameters and performance using neural network. The performance applied in this study is MAPE.

Step 5. Optimization: Obtain the hyper parameter combination with better performance using PSO. In this study, the goal of optimization is to minimize the MAPE of the CNN. 

Step 6. Verification: Verify the performance of the optimized result.

In this study, a simple neural network is applied for the model and particle swarm optimization (PSO) is adopted for optimization to compare with MR and the full-factorial searching algorithm [54].

### 3.2. Surface Roughness Estimation Using CNN

Data of milling are proposed by Wu et al. using a tungsten carbide milling cutter to cut S45C steel [34]. There are six single-axial accelerometers (Wilcoxon Research 785A) mounted on the spindle and vise for measuring X-axial, Y-axial, and Z-axial vibration signals. The signals are acquired using DAQ NI 9234 with 10 kHz of sampling frequency. The experimental setup can be found in [34]. The surface roughness is measured using Mitutoyo SV-C3200S4. The machining parameters and setup values are: spindle speed (rpm)—900, 1000, 1800, 1900, 2000, 2100, 2700, 3000 (rpm); feed rate—228, 240, 252, 320, 400, 420, 532, 560, 588 (mm/min); cutting depth—0.5, 0.6, 0.7, 0.8, 0.9, 1 (mm); and clamp force of vise—18, 30, 75 (N-m). There are a total of 153 data in the dataset. The complete data are available on the website [34].

**Figure 3:**
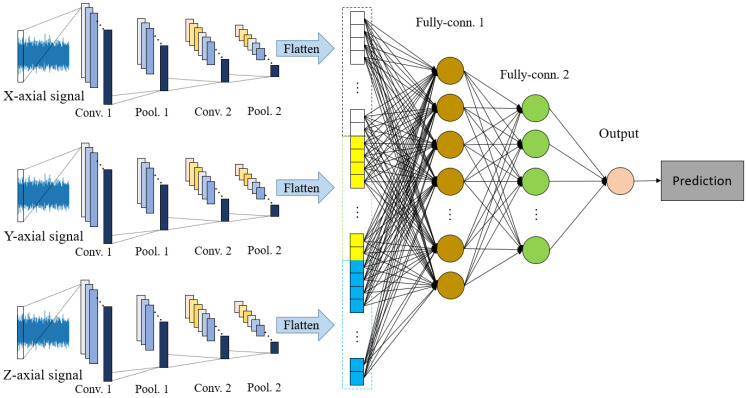
The sensors fusion structure for machining surface roughness estimation.

A one-dimensional CNN (1DCNN) with sensors fusion in parallel structure, shown in Figure 3, is applied for machining roughness estimation. The features of vibration signals in X, Y, Z directions are extracted separately. In order to obtain a CNN structure with better performance, the optimization for hyper parameters combination is applied [52]. The range of optimized hyper parameters and the structure of the CNN are selected as shown in Table 1. According to Table 1, there are six design factors: $F_{C}$ for the size of filters in convolutional layers, $F_{P}$ for the size of filters in pooling layers, $N_{C1}$ for the filter number in the first convolutional layer, $N_{C2}$ for the filter number in the second convolutional layer, $N_{F1}$ for the number of nodes in the first fully connected layer, and $N_{F2}$ for the number of nodes in the second fully connected layer. The feature extraction for three axial signals are the same. The performance of the model is assumed as a function of hyper parameters, which is represented as

(7) $$\mathrm{MAPE}=f_{MAPE}\left(F_{C},\;F_{P},\;N_{C1},\;N_{C2},\;N_{F1},\;N_{F2}\right)$$

According to UED [49], four levels are selected for all factors and the corresponding uniform layout applied here is $U_{28}\left(4^{6}\right)$, as shown as Table 2. The final experimental design is introduced in Table 3. The corresponding combinations of parameters and trained MAPE (average testing MAPE of corresponding experimental CNNs) are also introduced. Every structure has been tested three times and the average MAPEs are computed. The maximum epoch of each model is 700. In order to reduce the needed time for experiments, an early stop criterion is set up according to testing experiences: if the loss has not decreased for 15 epochs, the training process is stopped.

After the experiments, the function between hyper parameters and average testing MAPE is modeled using MR and NN for comparison. The performance of models, optimization results, and verifications are compared as follows. The data are normalized before modeling. 

At first, modeling using stepwise MR is obtained as

(8) $$\begin{array}{l} \mathrm{MAPE}=35.818395-1.215402F_{C}-0.428033F_{P}+0.758975N_{C1}+0.991905N_{C2}\\ \;\;\;\;\;\;+0.140401N_{F1}-0.224964N_{F2}+0.001241F_{C}N_{F2}+0.053019F_{C}N_{C2}\\ \;\;\;\;\;\;-0.046696F_{P}N_{C2}+0.01539F_{P}N_{F2}-0.070553N_{C1}N_{C2}-0.000967N_{C1}N_{F2}\\ \;\;\;\;\;\;-0.010024N_{C2}N_{F1}-0.00065N_{F1} \end{array}$$

The corresponding R-squared ($R^{2}$) of MR model is 0.9061 and the normalized root mean squared error (NRMSE) of MR is 0.0634. The objective function (fitness) is selected as the MAPE of each structure. The optimization target is to minimize the fitness. The hyper parameters combination optimized using the full-factorial searching algorithm are: $F_{C}=25$, $F_{P}=20$, $N_{C1}=20$, $N_{C2}=20$, $N_{F1}=100$, $N_{F2}=10$. The testing MAPE prediction of the MR model for the combination is 5.788%. The structure with the optimized hyper parameters combination has been trained three times. The testing MAPEs are shown in Table 4. The average MAPE is quite different to the prediction, with an error of 147.06%. The combination does not perform better compared to the experiments.

Then, an NN is applied to model the relation between factors and testing MAPE. The structure of NN is shown in Table 5. The initial learning rate is 0.005, and the optimizer is Adam. The *R*-squared ($R^{2}$) of NN is 0.9999999996 and the normalized root mean squared error (NRMSE) of the NN is $3.347\times 10^{-5}$. The hyper parameters combination optimized using the full-factorial searching algorithm are: $F_{C}=25$, $F_{P}=11$, $N_{C1}=18$, $N_{C2}=12$, $N_{F1}=100$, $N_{F2}=50$. The testing MAPE prediction of the NN model for the combination is 10.849%. The combination has also been trained three times. The testing MAPEs are shown in Table 6. The error between the average MAPE and prediction of the NN model is much smaller, with an error of 7.337%. The optimized structure improves the performance by 11.3%. The results show that modeling using NN can also create a better and more stable hyper parameters combination than the best hyper parameters set in the experiments. However, the structure, learning rate, and normalization affect the performance of modeling and optimized result a lot. A simple NN with a smaller learning rate is recommended in this case. Normalization is also necessary.

Herein, PSO is applied for optimization to compare with the full-factorial searching algorithm. Modeling using an NN is applied for comparison. The number of particles is selected as 250, and the number of iterations is set to be 3000. The reason for choosing this number of particles and iteration is to ensure the optimized result is the same as the result using the full-factorial searching algorithm. The weights of updating velocity are adjusted shown in Table 7. If the fitness of $P_{gbest}$ does not improve for 500 iterations, the optimization is stopped.

**Figure 4:**
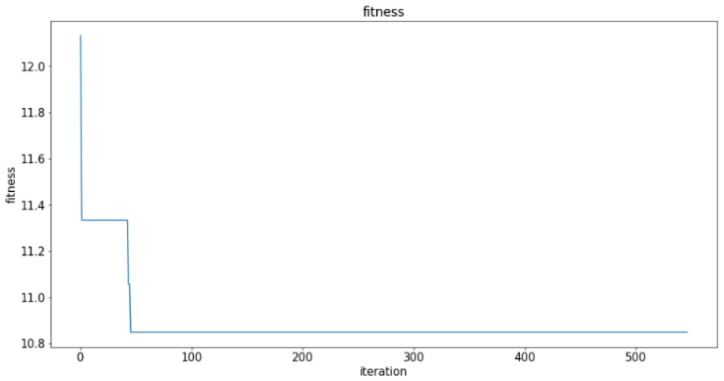
Fitness during optimization.

The fitness during optimizing using PSO is shown as Figure 4. The optimized result is the same as the full-factorial searching algorithm. Moreover, PSO takes 45.435 s to complete the process, while it takes 146.87 s for the full-factorial searching algorithm. If the number of particles and iterations are reduced according to the testing results, the time for optimization can be less than the previous experiment result. When the structure of the optimized CNN is more complex, the computing time for PSO and other optimization methods are much less compared to the time for the full-factorial searching algorithm.

## 4. Fault Diagnosis Applications

### 4.1. Classification of CWRU Bearing Data

Bearing data of CWRU [56] are discussed in many other studies for bearing fault classification [57,58,59]. The signals discussed in the study are collected by the accelerometer mounted at the drive end of motor. The sampling frequency is 12 kHz. The bearing statuses include normal bearings, bearings with inner ring faults, bearings with outer ring faults, and bearings with ball faults, which are human-made using an electrical-discharge machine (EDM). The statuses of bearings are labeled according to normal: 0; inner ring fault:1; outer ring fault: 2; and ball fault: 3, respectively. There are 64 data in the original dataset. In order to increase the number of data, sliding window is utilized to slice the signals into one-second signals. The length and the stride of window are 12,000 data points (1 s) and 3000 data points, respectively. The length of window is selected after considering the completeness of signals in the frequency domain and the testing results. Finally, there are 2368 data; 1657 data (70%) are chosen randomly as training data and the rest (30%) are applied as testing data.

(a) Bearing Faults Classification Using Vibration Signals

**Figure 5:**
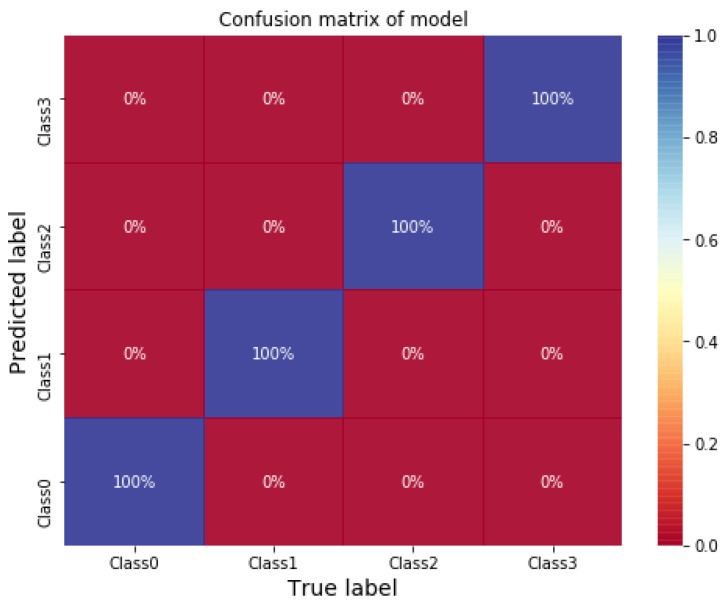
Confusion matrix of 1DCNN model for classifying CWRU bearing data. Reprinted from ref. [47].

Herein, we introduce the classification of bearing faults using 1DCNN with vibration signals as inputs. The selected structure of 1DCNN is introduced in Table 8. The initial learning rate is 0.001, and the optimizer is Adam. The average of training and testing accuracy of the model are both 100% after testing three times using different training data. The confusion matrix of the model predicting testing data is shown in Figure 5. The result shows that 1DCNN can provide excellent performance using vibration signals as inputs directly for classification. The classifying time of 1DCNN using NVIDIA Tesla V100 32 GB GPU is 0.00133 s per data.

(b) Bearing Faults Classification Using STFT Time-Frequency Spectra

**Figure 6:**
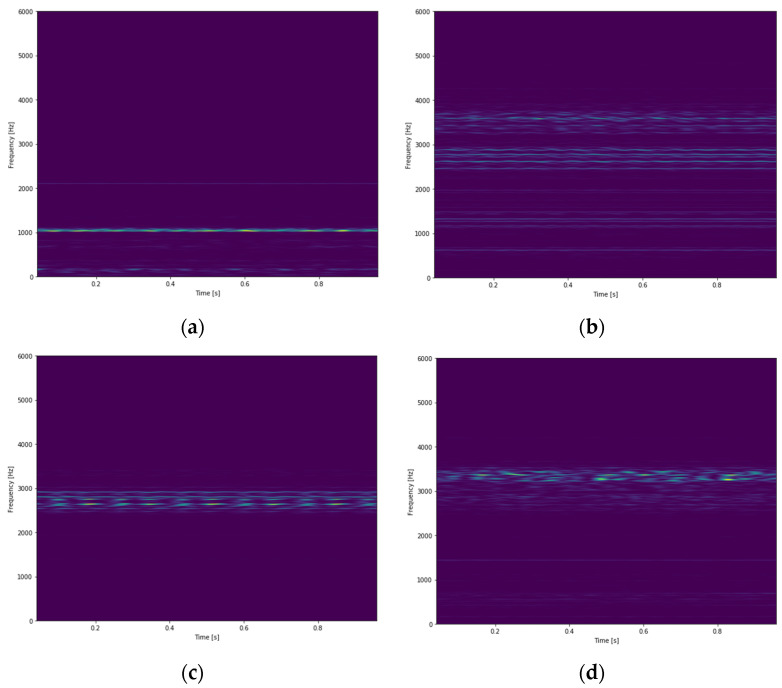
STFT time-frequency spectra of different bearing conditions, (**a**) a normal bearing; (**b**) a bearing with inner ring fault; (**c**) a bearing with outer ring fault; (**d**) a bearing with ball fault [47].

**Figure 7:**
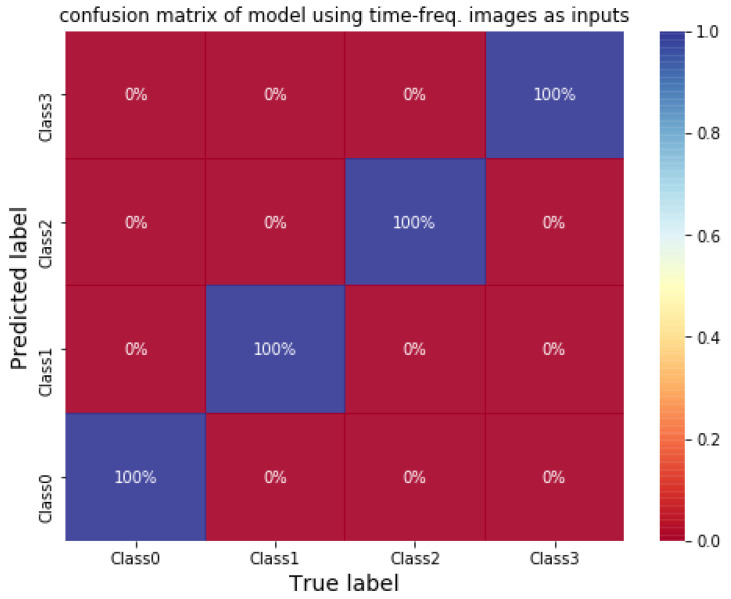
Confusion matrix of CNN for classifying bearing faults [47].

The time-frequency spectra after STFT of different bearing conditions are shown in Figure 6. A 2DCNN is applied to classify the bearing faults. The structure of the CNN is shown as Table 9. The initial learning rate is 0.001 with the Adam optimizer. The average of training and testing accuracy are both 100% after testing three times. The confusion matrix of the model for testing data is shown as Figure 7. The result shows that 2DCNN can also be applied for the classification of bearing faults with great performance. The inputs of 2DCNN can be other types of two-dimensional arrays, e.g., time-frequency spectra using wavelet transform. The transformation time using STFT is 0.75258 s per data, and the classifying time of 2D CNN using NVIDIA Tesla V100 32 GB GPU is 0.00419 s per data. Classification using 2DCNN takes more time due to the input size of the model. 1DCNN uses raw signals as inputs; the input size is 12,000 × 1. 2DCNN uses STFT time-frequency spectra as inputs; the input size is 434 × 558 × 3.

### 4.2. Classification of Tool Wear Using STFT Time-Frequency Spectra

**Figure 8:**
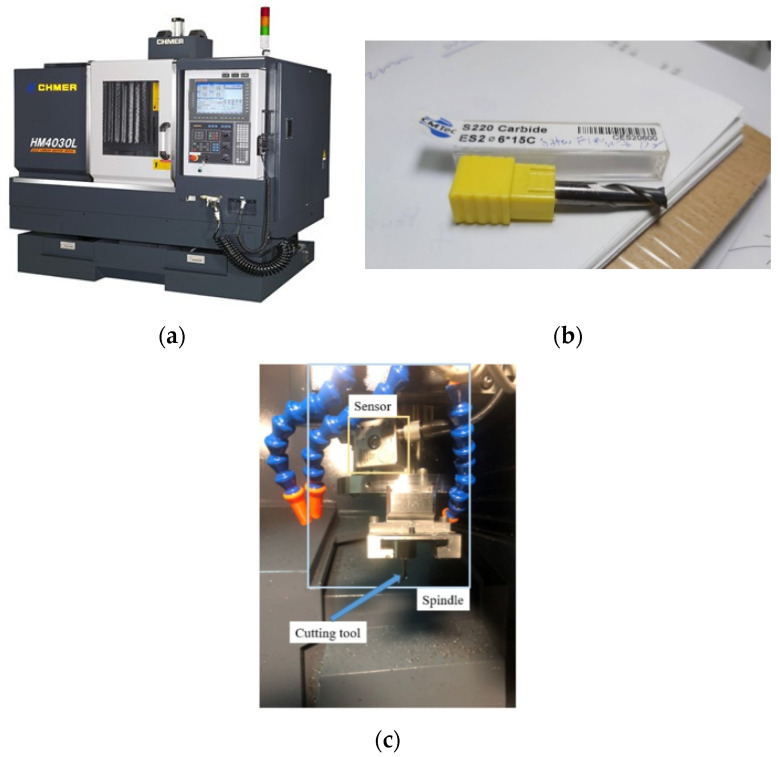
Experimental setup for tool wear monitoring, (**a**) CHMER HM4030L tri-axial milling machine; (**b**) tungsten carbide milling cutter for the experiments; (**c**) setup of CTC AC230 on the spindle.

The experimental setup is introduced in Figure 8; the tool wear data of a tri-axial milling machine (CHMER HM4030L, Figure 8a) are applied in the study. The machine tools are a tungsten carbide milling cutter with two blades, as shown in Figure 8b. The diameter of the cutters is 6 mm. The work-pieces are S45C steel. The tri-axial accelerometer (CTC AC230) is mounted on the spindle, as shown in Figure 8c. The vibration signals are acquired using DAQ NI PCIe-6361 with 100 kHz of sampling frequency. The tool wear is measured using a Deryuan RS-500 industrial camera with ImageJ and PhotoImpact for image processing. The tool worn criteria is selected as 0.4 mm according to ISO.

**Figure 9:**
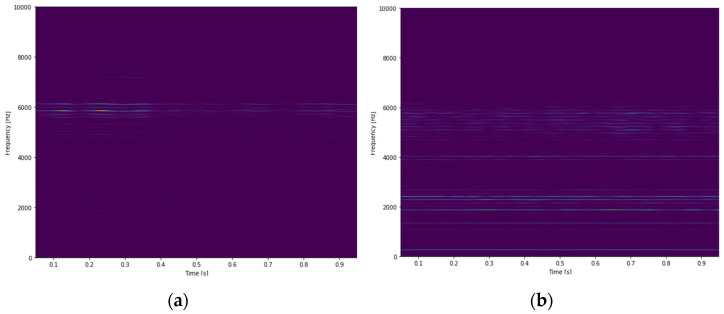
STFT time-frequency spectra of tools under different conditions, (**a**) an unworn tool; (**b**) a worn tool.

**Figure 10:**
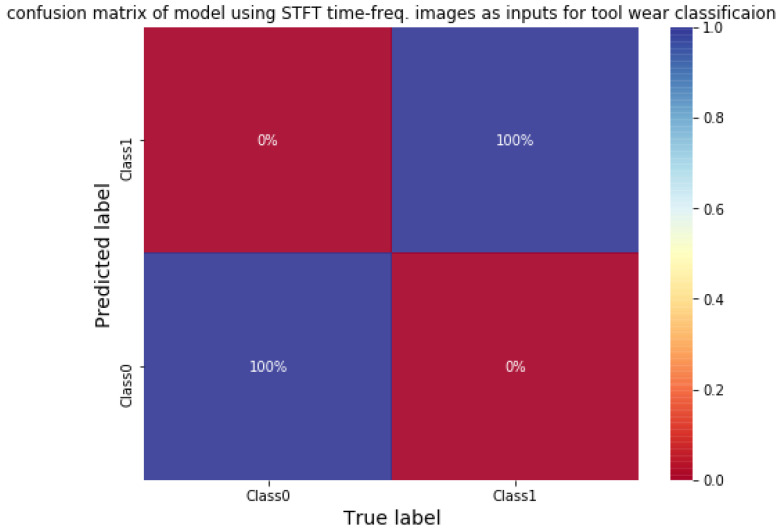
Confusion matrix of CNN for classifying tool wear.

A 2DCNN with a small structure (shown in Table 10) is adopted for classifying tool wear using STFT time-frequency spectra. The vibration signals are sliced using sliding window to increase the size of data. The length and stride of window is 100,000 data points (1 s) and 30,000 data points, respectively. The STFT time-frequency spectra using Y-axial vibration signals of an unworn tool and a worn tool are shown in Figure 9. There are a total of 742 data; half of the data are selected randomly as training data, and the rest are testing data. Firstly, the classification model is trained. The initial learning rate is 0.001 with the Adam optimizer. The average training and testing accuracy are both 100% after testing three times. The confusion matrix of the CNN model using testing data is shown in Figure 10. The result shows that 2DCNN can be applied for not only bearing faults classification but also other classified problems in vibration signals analysis. 

## 5. Conclusions

In this study, vibration signals analysis using CNN has been discussed, including an improved optimization method for the structure of a CNN, 1DCNN and 2DCNN with raw signals and STFT images, respectively. The experimental results were introduced to illustrate that the CNN can be applied for both prediction and classification. In regression application, a 1DCNN with parallel feature extracting structure was applied to estimate machining roughness. The optimization of the CNN structure was also introduced and used to demonstrate the effectiveness of the proposed approach to obtain a structure with better performance. The most important factor in optimizing the structure of CNN is to choose the correct method and level for the experimental design. The level can be comprehended as the resolution experiments. If the level is too large, the number of experiment results is too little to represent the real situation. On the other hand, the cost of time will be enhanced due to the large number of experiments. Other experimental design can also be applied; for instance, the Taguchi method. In classifications, 1DCNN and 2DCNN are applied according to the inputs. Both 1DCNN and 2DCNN provide excellent performance. The results also show that CNN can extract features in vibration signals and time-frequency spectra automatically. While using raw signals as inputs, the length of signal must be long enough to ensure the information of the signal is complete. If time-frequency spectra are utilized as inputs, the resolution of STFT affects the model since time-frequency spectra show the distribution of frequency with respect to time. If the resolution is not appropriate, the information in the frequency domain will be reduced and influence the performance of model.

## Figures and Tables

**Table 1:** Hyper parameters of CNN for machining surface roughness estimation.

Layers	Filter Size	Stride	Number of Filters or Nodes	Activation Function
Conv. 1 (X, Y, Z)	$F_{C}$ (16~25)	2	$N_{C1}$ (11~20)	ReLU
Pool. 1 (X, Y, Z)	$F_{P}$ (11~20)			
Conv. 2 (X, Y, Z)	$F_{C}$ (16~25)	2	$N_{C2}$ (11~20)	ReLU
Pool. 2 (X, Y, Z)	$F_{P}$ (11~20)			
Flatten				
Fully connected 1			$N_{F1}$(10~100)	ReLU
Fully connected 2			$N_{F2}$(10~100)	ReLU
Output			1	None

**Table 2:** $U_{28}\left(4^{6}\right)$ uniform layout.

Experiment Index	Factors					
	$F_{C}$	$F_{P}$	$N_{C1}$	$N_{C2}$	$N_{F1}$	$N_{F2}$
1	1	3	2	3	4	3
2	4	4	3	2	4	2
3	2	3	3	3	3	2
4	1	2	1	4	4	2
5	2	2	3	1	2	3
6	1	4	1	1	1	3
7	3	1	3	4	2	1
8	3	3	3	1	1	4
9	1	2	3	2	1	1
10	3	4	2	2	2	3
11	4	2	4	2	3	3
12	2	1	1	3	1	3
13	4	1	3	4	4	3
14	2	4	4	1	4	1
15	1	1	4	3	2	2
16	3	1	1	2	1	2
17	3	2	1	1	3	4
18	4	3	4	1	2	2
19	1	3	4	4	3	4
20	4	4	1	3	3	4
21	4	2	2	3	2	4
22	4	3	2	4	1	1
23	3	2	4	3	4	1
24	2	3	1	2	2	1
25	2	1	2	2	4	4
26	1	1	2	1	3	1
27	3	4	2	4	3	2
28	2	4	4	4	1	4

**Table 3:** Experimental design of CNN structure for estimating machining roughness and average testing MAPE of corresponding experimental CNNs.

Experiment Index	$F_{C}$	$F_{P}$	$N_{C1}$	$N_{C2}$	$N_{F1}$	$N_{F2}$	Parameters	Avg. Testing MAPE (%)
1	16	17	14	17	100	70	60,230	14.35
2	25	20	17	14	100	40	44,399	13.57
3	19	17	17	17	70	40	49,055	16.00333333
4	16	14	11	20	100	40	87,362	18.42666667
5	19	14	17	11	40	70	30,533	18.3
6	16	20	11	11	10	70	8903	23.83333333
7	22	11	17	20	40	10	69,734	25.16
8	22	17	17	11	10	100	17,399	23.11333333
9	16	14	17	14	10	10	17,504	24.25666667
10	22	20	14	14	40	70	25,325	19.11
11	25	14	20	14	70	70	60,053	15.17333333
12	19	11	11	17	10	70	21,911	25.44
13	25	11	17	20	100	70	148,127	11.33666667
14	19	20	20	11	100	10	31,394	18.82666667
15	16	11	20	17	40	40	57,872	18.46333333
16	22	11	11	14	10	40	19,436	21.03
17	22	14	11	11	70	100	43,769	18.4
18	25	17	20	11	40	40	29,054	16.59333333
19	16	17	20	20	70	100	61,151	13.68333333
20	25	20	11	17	70	100	40,055	18.50333333
21	25	14	14	17	40	100	45,674	18.52333333
22	25	17	14	20	10	10	26,483	19.17333333
23	22	14	20	17	100	10	86,192	16.02333333
24	19	17	11	14	40	10	23,381	19.54333333
25	19	11	14	14	100	100	102,155	15.81
26	16	11	14	11	70	10	52,820	28.36333333
27	22	20	14	20	70	40	43,457	15.21333333
28	19	20	20	20	10	100	28,271	18.87666667

**Table 4:** Testing MAPEs of the optimized hyper parameters combination using MR model.

Test MAPE 1	Test MAPE 2	Test MAPE 3	Avg. MAPE	Standard Deviation
15.74%	13.97%	13.19%	14.3%	1.090%

**Table 5:** Structure of NN for modeling the function between factors and testing MAPE.

Layer	Nodes	Activation Function	Bias
Input	6	None	None
Hidden 1	12	Sigmoid	None
Output	1	None	Yes
Total parameters	85		

**Table 6:** Testing MAPEs of the optimized hyper parameters combination using NN model.

Test MAPE 1	Test MAPE 2	Test MAPE 3	Avg. MAPE	Standard Deviation
11.04%	10.68%	8.44%	10.053%	1.150%

**Table 7:** Adjustment details of weights while updating velocity.

Weights of Updating Velocity	Range of Values	Adjustment of Weights
*w*	0.1~2	Decrease while the iteration increases.
$c_{1}$	0.1~2	Decrease while the iteration increases.
$c_{2}$	0.1~2	Increase while the iteration increases.

**Table 8:** Structure of 1DCNN for bearing faults classification using vibration signals.

Layer	Filter Size	Stride	Number of Filters or Nodes	Activation Function
Conv. 1	30	1	8	ReLU
Pool. 1	4			
Conv. 2	30	1	16	ReLU
Pool. 2	4			
Conv. 3	30	1	32	ReLU
Pool. 3	4			
Conv. 4	30	1	64	ReLU
Pool. 4	4			
Flatten				
Fully Conn. 1			128	ReLU
Fully Conn. 2			32	ReLU
Output			4	Softmax
Total parameters	388,488			

**Table 9:** Structure of CNN for classifying bearing faults.

Layer	Filter Size	Stride	Number of Filters or Nodes	Activation Function
Conv. 1	9×9	2×2	4	ReLU
Conv. 2			8	ReLU
Pool. 2	4×4			
Conv. 3	4×4	2×2	16	ReLU
Conv. 4			32	ReLU
Pool. 4	2×2			
Flatten				
Fully Conn. 1			64	ReLU
Fully Conn. 2			32	ReLU
Output			4	Softmax
Total parameters	63,622			

**Table 10:** Structure of CNN for classifying tool wear.

Layer	Filter Size	Stride	Number of Filters or Nodes	Activation Function
Conv. 1	9×9	2×2	4	ReLU
Conv. 2			8	ReLU
Pool. 2	4×4			
Conv. 3	4×4	2×2	16	ReLU
Conv. 4			32	ReLU
Pool. 4	2×2			
Flatten				
Fully Conn. 1			64	ReLU
Fully Conn. 2			32	ReLU
Output			2	Softmax
Total parameters	28,360			

## Data Availability

The used data of bearing fault can be found in Case Western Reserve University Bearing Data Center. Available online: http://csegroups.case.edu/bearingdatacenter/pages/wel-come-case-western-reserve-university-bearing-data-center-website (accessed on 10 March 2019).
